# Silencing of long-non-coding RNA ANCR suppresses the migration and invasion of osteosarcoma cells by activating the p38MAPK signalling pathway

**DOI:** 10.1186/s12885-019-6335-4

**Published:** 2019-11-14

**Authors:** Bo Liu, Hongyan Zhao, Lili Zhang, Xuefeng Shi

**Affiliations:** 1 The Third Department of Orthopedics, The No. 4 Hospital of Jinan, No. 50, Shifan Road, Tianqiao District, Jinan City, 250031 Shandong Province China; 2 Department of Community Section, The First People’s Hospital of Jinan, No. 132, Daminghu Road, Lixia District, Jinan City, 250011 Shandong Province China; 3 Department of Gynecology, The No. 4 Hospital of Jinan, No. 50, Shifan Road, Tianqiao District, Jinan City, 250031 Shandong Province China; 4 Department of Orthopedic Trauma & Hand and Foot Surgery, Jinan Central Hospital Affiliated to Shandong University, No. 105, Jiefang Road, Jinan City, 250013 Shandong Province China

**Keywords:** Osteosarcoma, Long non-coding RNA ANCR, p38MAPK signalling pathway, Migration, Invasion

## Abstract

**Background:**

Osteosarcoma (OS) is a malignancy of the bone that has no clearly identified prognostic factors for diagnosis. In this study, we evaluated the regulatory role of long non-coding RNA (lncRNA) ANCR on the migration and invasion of OS cells as well as the possible mechanism involving the p38MAPK signalling pathway.

**Methods:**

ANCR expression was determined in OS tissues and OS cell lines (MG-63, S1353, U2OS, and UMR-106) by qRT-PCR. It was observed that ANCR was down-regulated in MG-63 and U2OS cells by 48 h of siRNA-ANCR (si-ANCR) transfection. The proliferation of transfected cells was determined using the CCK-8 and the EdU assays. The migration and invasion of transfected cells were determined by the Transwell assay. The expression of E-cadherin, N-cadherin, and phosphorylated p38MAPK (p-p38MAPK) proteins was determined by Western blot. In addition, combinatorial treatment of cells with si-ANCR + SB203580 (p38MAPK inhibitor) was performed to investigate the association between ANCR and MAPK signalling in OS cells.

**Results:**

ANCR was up-regulated in OS cells and tissues. ANCR silencing significantly inhibited the proliferation rate, decreased the percentage of migration and invasion cells, down-regulated N-cadherin, and up-regulated E-cadherin and p-p38MAPK in MG-63 and U2OS cells. Inhibition of the p38MAPK signalling pathway (SB203580) in MG-63 and U2OS cells rescued si-ANCR-induced inhibition of cell migration and invasion.

**Conclusions:**

Silencing of ANCR inhibited the migration and invasion of OS cells through activation of the p38MAPK signalling pathway.

## Background

Osteosarcoma (OS) is an aggressive bone cancer that commonly occurs in children and adolescents accounting for about more than 60% of the malignant bone tumours in patients under the age of 20 years [1, 2]. OS mainly originates from active regions in the bone, including lower femur, upper tibia, and knee joint [3, 4]. Common manifestations in patients with OS include the onset of pain and bone swelling, and the pain is strong enough to wake a patient up [5]. Statistics showed that the improvement of surgical techniques as well as the use of multiple doses and dose-intensive chemotherapy increased the 5-year survival of patients with localized OS to 60%, but this outcome still needs to be improved [6]. Evidence has proved that patients with OS are at high risk of local invasion and early systemic metastasis [7]. Besides, traditional OS is accompanied by diverse genomic aberrations and gene expression changes at the molecular level [8]. Therefore, finding new therapeutic targets and methods has become an important challenge in OS treatment.

Long-non-coding RNAs (lncRNAs) are important regulatory molecules that are involved in diverse biological processes [9]. Significantly altered expression of lncRNAs is observed in OS tissues and cells [10]. Anti-differentiation non-coding RNA (ANCR) containing 855 base pairs maintains the undifferentiated state of epidemic stem cells and osteoblast cells [11]. ANCR expression is usually decreased during the differentiation process. Previous evidence has revealed that ANCR can influence the growth of periodontal ligament stem cells as well as the osteogenic differentiation [12]. Noteworthy, ANCR is also a key regulator in OS pathogenesis. It has been proved that ANCR knockdown in U2OS cells inhibits both cell viability and colony formation ability [13]. ANCR knockdown in MG-63 and UMR-106 cells inhibits cell proliferation, migration, and invasion [14]. P38 mitogen-activated protein kinase (p38MAPK), a kinase present in postsynaptic dendrites, is involved in the regulation of cytoskeletal reorganization [15, 16]. p38MAPK can be activated by the upstream kinases, MKK3 and MKK6, and activation of p38MAPK regulates cell migration and metastasis [17]. Interestingly, accumulating evidence has confirmed the important role of the MAPK signalling pathway in OS [18, 19]. For example, capsaicin inhibits viability and colony formation, and induces the cell cycle arrest at G0/G1 phase in three OS cell lines, MG63, 143B, and HOS through activating the MAPK signalling pathway [20]. Escin induces the apoptosis and autophagy of HOS and Saos-2 cells by activating the MAPK signalling pathway [21]. However, whether the regulatory mechanism of ANCR in OS cells is associated with the MAPK signalling pathway remains unclear. Thus, we evaluated the specific effects of lncRNA-ANCR on cell migration and invasion as well as the potential mechanism involving the MAPK signalling pathway in OS cells.

## Methods

### Sample collection

A total of 61 patients with OS (35 males and 26 females, aged 14~54 years, median age 27 years) were screened from the orthopaedics department of our hospital between January 2012 and December 2017. OS and adjacent normal tissues (adjacent mucosa) were collected from patients who underwent surgery. All tumour specimens were pathologically confirmed and preserved in liquid nitrogen within 30 min after surgery. This study was approved by the ethics committee of Jinan Central Hospital Affiliated to Shandong University. Informed consent was obtained from all subjects as well as parental consent for subjects aged less than 18 years.

### Cell culture

OS cell lines, MG-63 (#TCHu124), SW1353 (#TCHu128), U2OS (#TCHu88), and UMR-106 (#TCR11) as well as the osteoblast cell line, hFOB1.19 (#GNHu14) were purchased from Cell Bank of the Chinese Academy of Sciences (Shanghai, China). Cells were cultured in Dulbecco’s modified Eagle’s Medium (DMEM) (HyClone) containing 10% foetal bovine serum (Gibco, Grand Island, NY, USA) in a constant temperature incubator at 37 °C and 5% CO_2_. Cells were passaged at the ratio of 1:3 at 90% confluence.

### Quantitative real time PCR (qRT-PCR)

Total RNA was extracted from specific tissues and cells using TRIzol total RNA extraction kit. cDNA was synthesised by reverse transcription. qRT-PCR was performed on a ABI7500 PCR instrument (ABI, Austin, TX, USA) using the following conditions: 95 °C for 10 min, 50 cycles of 95 °C for 15 s, 60 °C for 1 min, and 72 °C for 40 s. The primer sequences are shown in Table 1. 18S rRNA was used as an internal control and the 2^-ΔΔCt^ method was used to analyse the data. This experiment was repeated five times.

**Table 1:** Primer sequences of RT-PCR

	Primer sequences
LncRNA ANCR	5′-GACATTTCCTGAGTCGTCTTCGAACGGAC-3′
	5′-TAGTGCGATTTAGAGCTGTACAAGTTTC-3′
18r RNA	5′-ACACGGACAGGATTGACAGA-3′
	5′-GGACATCTAAGGGCATCACA-3′

*RT-PCR* Reverse transcription-polymerase chain reaction

### Cell grouping

Cells were transfected with ANCR siRNA (F: 5′-GATCCCCGAGCTAGAGCAGTGACAATTTCAAGAGAATTGTCACTGCTCTAGCTCTTTTTC-3′; R: 5′-TCGAGAAAAAGAGCTAGAGCAGTGACAATTCTCTTGAAATTGTCACTGCTCTAGCTCGGG-3′) (si-ANCR group) or siRNA negative control (F: 5′-GATCCCCTTCTCCGAACGTGTCACGTTTCAAGAGAACGTGACACGTTCGGAGAATTTTTC-3′; R: 5′-TCGAGAAAAATTCTCCGAACGTGTCACGTTCTCTTGAAACGTGACACGTTCGGAGAAGGG-3′) (NC group) using the Lipofectamine™ 2000 Transfection reagent (Invitrogen, Carlsbad, CA, USA). Cells in the si-ANCR + SB203580 group were transfected with ANCR siRNA and SB203580 (p38MAPK inhibitor SB203580, Merck, NJ, USA; final concentration, 50 μmol/L). Untransfected cells were used as the blank group. Cells were used for further assays at 48 h post-transfection.

### Cell counting kit-8 (CCK-8) assay

CCK-8 assay was performed using the CCK-8 kit (Beyotime, Shanghai, China) as previously described [22]. The OD_450_ was determined with a microplate reader (Bio-Rad, Hercules, CA, USA). Six duplicated wells were set for this experiment.

### EdU proliferation assay

Cells were inoculated into 6-well plates (3 × 10^3^ cells/well) and cultured for 24 h. After 30 min of fixation with 4% formaldehyde, and 10 min of treatment with 0.5% Triton X-100 for 10 min, cells were stained with EdU (red) for 1 h, and counter-stained with Hoechst33342 (blue) for 30 min. The percentage of EdU positive staining was considered as the cell proliferation rate. Three duplicated wells were set for this experiment.

### Transwell assay

Transwell assay was performed by using a Transwell chamber (BD, USA) as previously described [23]. Cells passing into the lower chamber were counted in the upper, low, left, right, and middle fields of vision under a microscope (Olympus, Japan).

### Western blot analysis

Total proteins were isolated from cells, separated by 10% SDS-polyacrylamide gel electrophoresis and transferred into a Polyvinylidene Fluoride (PVDF) membrane (Millipore, Billerica, MA, USA). After 1 h of blocking with 0.5% dried skimmed milk at 25 °C, the membrane was incubated with the primary antibody at 4 °C overnight. The primary antibodies included antibodies against p38MAPK (ab32142, 1:100), p-p38MAPK (ab47363, 1:100), E-cadherin (ab1416, 1:50), N-cadherin (ab18203, 1:300), and GAPDH (ab9385, 1:5000). Subsequently, the membrane was incubated with sheep anti-rabbit second antibody for 1 h. Protein bands were developed with a chemiluminescent reagent, transformed to grey and quantified using an imaging software. The relative expression of the target protein was standardized with respect to GAPDH that was used as an internal reference (grey value).

### Statistical analysis

Data were processed with SPSS 21.0. Data normality was analysed by the Kolmogorov-Smirnov test. The data were expressed as mean ± standard deviation. Student *t* test was conducted to compare two groups. Single factor analysis of variance (ANOVA) was conducted to compare multiple groups. The non-parametric Kruskal-Wallis test was used to analyse the skewness of data, and Dunn’s test of multiple comparisons was performed. *P* < 0.05 represented statistically significant.

## Results

### ANCR is up-regulated in OS

**Fig. 1:**
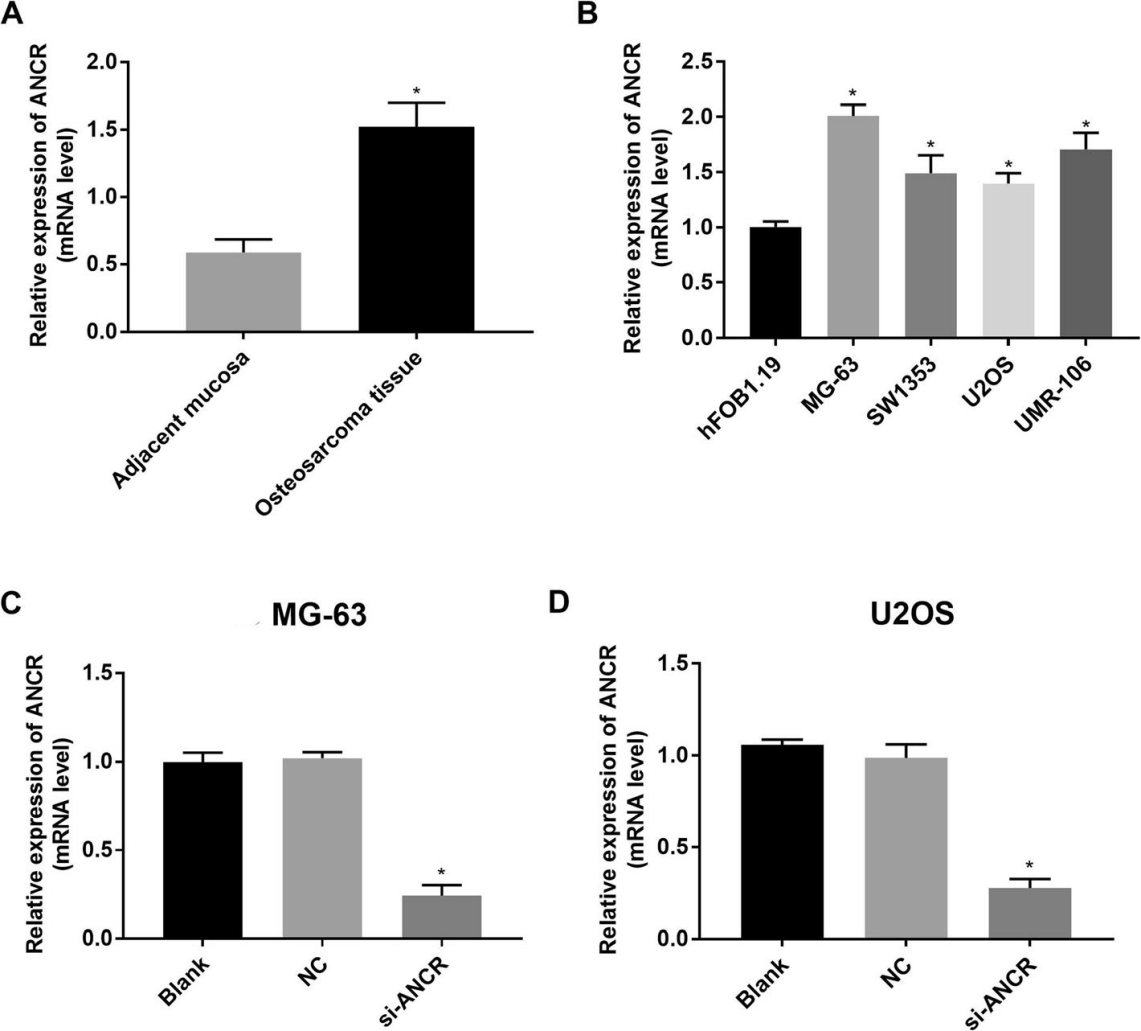
The expression of lncRNA-ANCR in osteosarcoma (OS) tissues and cells detected by quantitative real time PCR. **a** relative expression of ANCR in OS tissue and adjacent normal tissues (adjacent mucosa) at the mRNA level (*N* = 61); **b** relative expression of ANCR in four OS cell lines, including MG-63, SW1353, U2OS and UMR-106, as well as osteoblast cell line hFOB1.19 at the mRNA level (*N* = 5); **c** relative expression of ANCR in transfected MG-63 cells at the mRNA level (*N* = 5); **d** relative expression of ANCR in transfected U2OS cells at the mRNA level (*N* = 5). si-ANCR, cells transfected with siRNA-ANCR for 48 h; NC, cells transfected with siRNA negative control for 48 h; Blank, cells without transfection. *, *P* < 0.05 vs. adjacent mucosa (**a**), hFOB1.19 cells (**b**), as well as NC and Blank (**c** and **d**)

ANCR expression in OS tissues was significantly higher than that in adjacent normal tissues (adjacent mucosa) (Fig. 1a). Significantly higher ANCR expression was observed in OS cell lines (MG-63, SW1353, U2OS, and UMR-106) than that in hFoB1.19 cells (*P* < 0.05) (Fig. 1b). Among the four OS cell lines, MG-63 cells (relatively high ANCR expression) and U2OS cells (relatively low ANCR expression) were used for further assays. ANCR expression was significantly down-regulated in both MG-63 and U2OS cells at 48 h of si-ANCR transfection (*P* < 0.05). The transfection of NC in MG-63 and U2OS cells did not influence ANCR expression (Fig. 1c and d).

### Silencing of ANCR inhibits the proliferation of OS cells

**Fig. 2:**
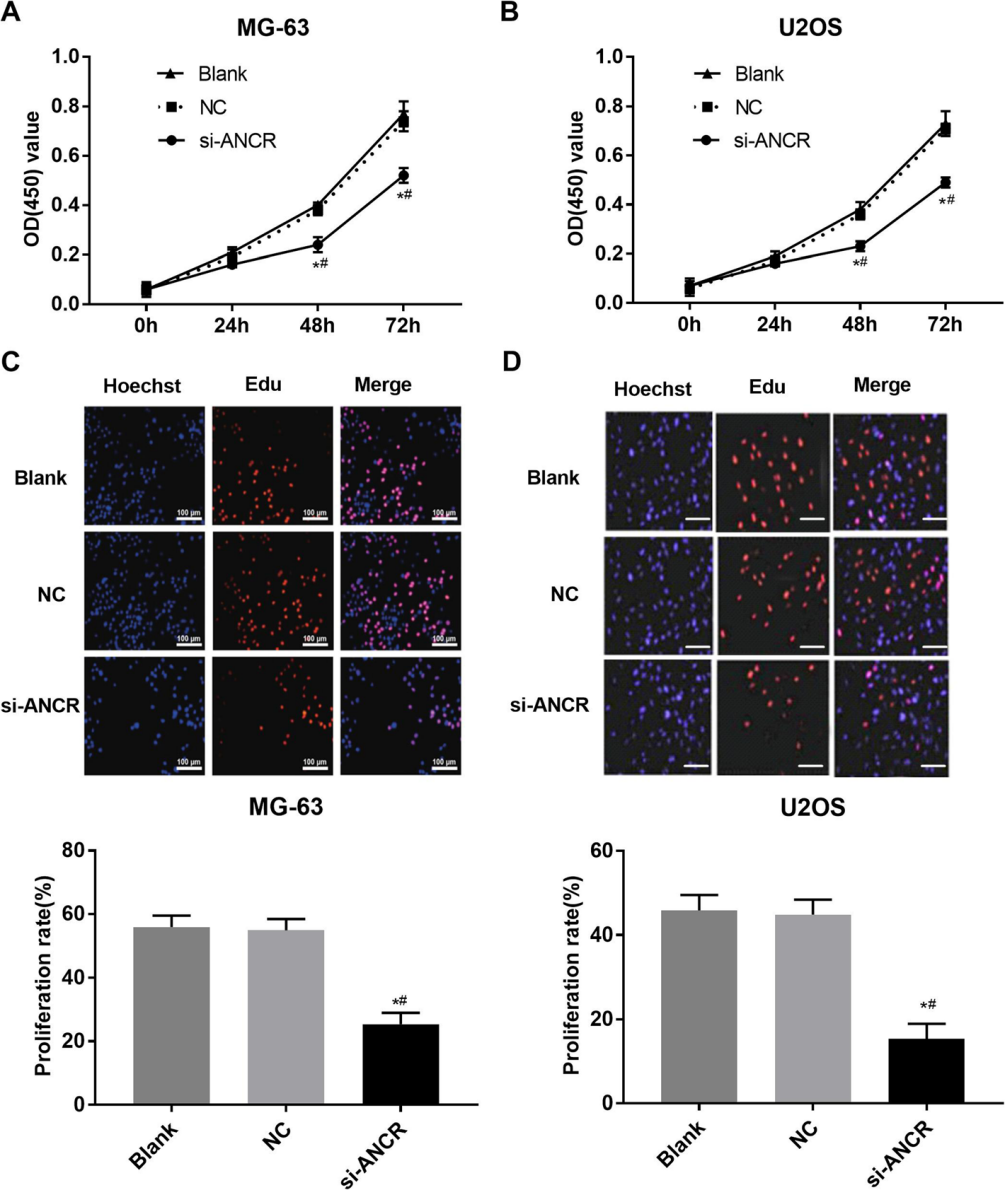
The proliferation of MG-63 and U2OS cells. **a** OD (450) values of MG-63 cells detected by CCK-8 assay (*N* = 6); **b** OD (450) values of U2OS detected by CCK-8 assay (*N* = 6); **c** the proliferation rat (%) of transfected MG-63 cells detected by EdU assay (*N* = 3); **d** the proliferation rat (%) of transfected U2OS cells detected by EdU assay (*N* = 3). si-ANCR, cells transfected with siRNA-ANCR for 48 h; NC, cells transfected with siRNA negative control for 48 h; Blank, cells without transfection. *, *P* < 0.05 vs. NC; #, *P* < 0.05 vs. Blank

The CCK-8 assay was used to determine the viability of MG-63 and U2OS cells. The OD_450nm_ was significantly increased in both MG-63 and U2OS cells in a time- and dose-dependent manner. After 48 and 72 h of culturing, the si-ANCR group exhibited significantly lower OD than the blank and NC groups (*P* < 0.05) (Fig. 2a and b). Compared with the blank and NC groups, the proliferation rate in the si-ANCR group was significantly decreased, as seen the by EdU assay (Fig. 2c and d). No significant differences were observed between blank and NC groups with respect to the OD and proliferation rate (Fig. 2a-d). The above findings indicate that silencing of ANCR can inhibit the proliferation of OS cells.

### Silencing of ANCR suppresses the migration and invasion of OS cells

**Fig. 3:**
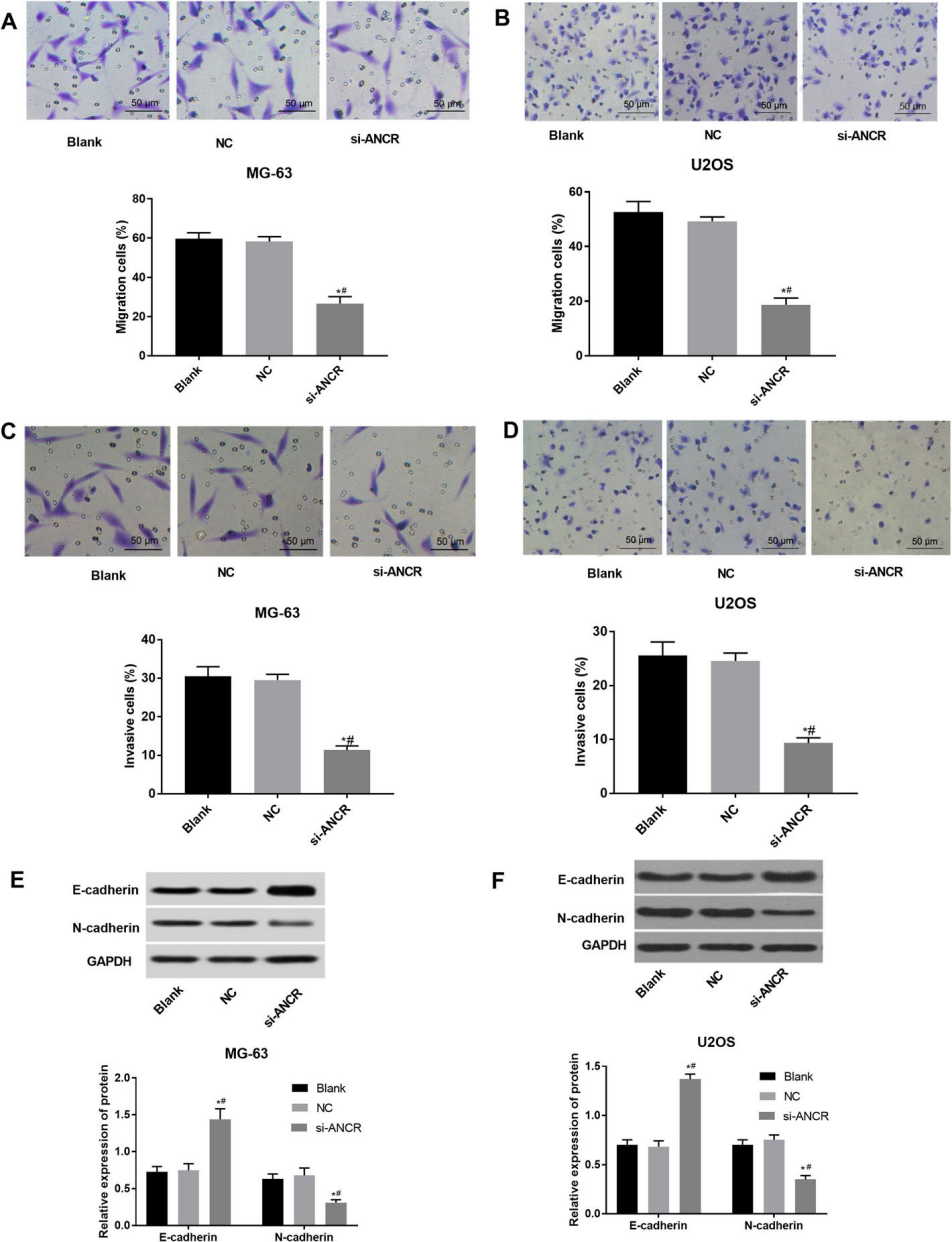
The migration and invasion of MG-63 and U2OS cells detected by Transwell assay and Western blot. **a** the percentage of migratory MG-63 cells (%) (*N* = 3); **b** the percentage of migratory U2OS cells (%) (*N* = 3); **c** the percentage of invasive MG-63 cells (%) (*N* = 3); **d** the percentage of invasive U2OS cells (%) (*N* = 3); **e** the expression of E-cadherin and N-cadherin in MG-63 cells at the protein level (*N* = 3); **f** the expression of E-cadherin and N-cadherin in U2OS cells at the protein level (*N* = 3). si-ANCR, cells transfected with siRNA-ANCR for 48 h; NC, cells transfected with siRNA negative control for 48 h; Blank, cells without transfection. *, *P* < 0.05 vs. NC; #, *P* < 0.05 vs. Blank

The Transwell assay was used to determine cell migration and invasion in the different cell groups. The percentages of cell migration (Fig. 3a and b) and invasion (Fig. 3c and d) were significantly lower in the si-ANCR group than that in the blank and NC groups. The levels of cell migration and invasion-related proteins were further determined by western blot. The si-ANCR group showed up-regulated E-cadherin and down-regulated N-cadherin at the protein level relative to the blank and NC groups (*P* < 0.05) (Fig. 3e and f). The migration and invasion were not significantly influenced by NC transfection in MG-63 and U2OS cells (Fig. 3a-f). The above findings indicate that ANCR silencing can suppress the migration and invasion of OS cells.

### ANCR silencing activated the p38MAPK signalling pathway in OS cells

**Fig. 4:**
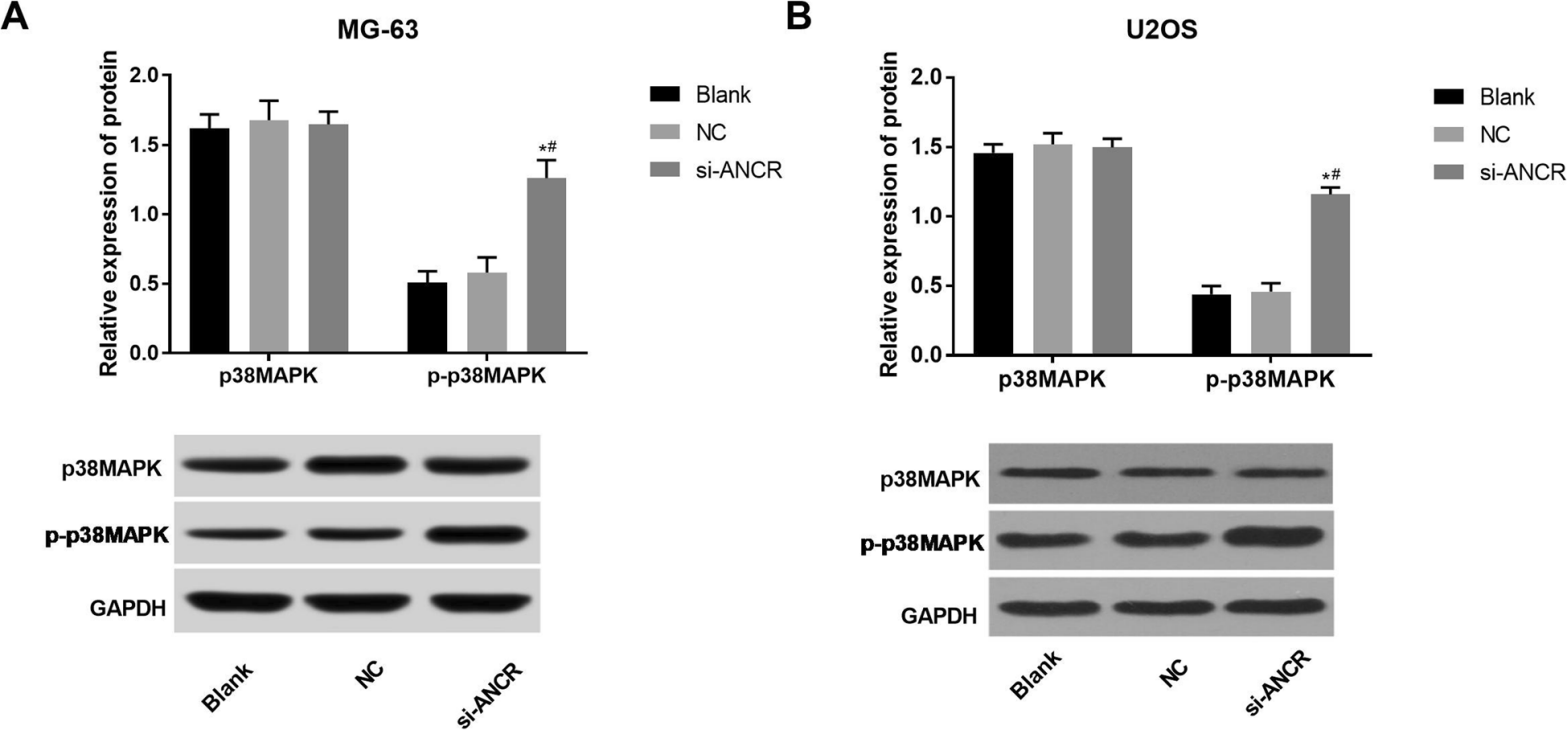
The expression of p38MAPK, and phosphorylated p38MAPK (p-p38MAPK) in MG-63 and U2OS cells detected by Western blot. **a** relative expression of p38MAPK and p-p38MAPK in MG-63 cells at the protein level (*N* = 3); **b** relative expression of p38MAPK and p-p38MAPK in U2OS cells at the protein level (*N* = 3). si-ANCR, cells transfected with siRNA-ANCR for 48 h; NC, cells transfected with siRNA negative control for 48 h; Blank, cells without transfection. *, *P* < 0.05 vs. NC; #, *P* < 0.05 vs. Blank

The expression of the p-p38MAPK protein in the si-ANCR group was significantly higher than that in the blank and NC groups (*P* < 0.05), while the expression of the p38MAPK protein was not significantly changed (Fig. 4a and b). In addition, no significant differences were observed between the blank and NC groups with respect to the expression of p-p38MAPK and p38MAPK (Fig. 4a and b). The above findings indicate that ANCR silencing may influence the biological function of OS cells by activating the p38MAPK signalling pathway.

### Inhibition of the p38MAPK signalling pathway eliminated the inhibitory effects of si-ANCR on the migration and invasion of OS cells

**Fig. 5:**
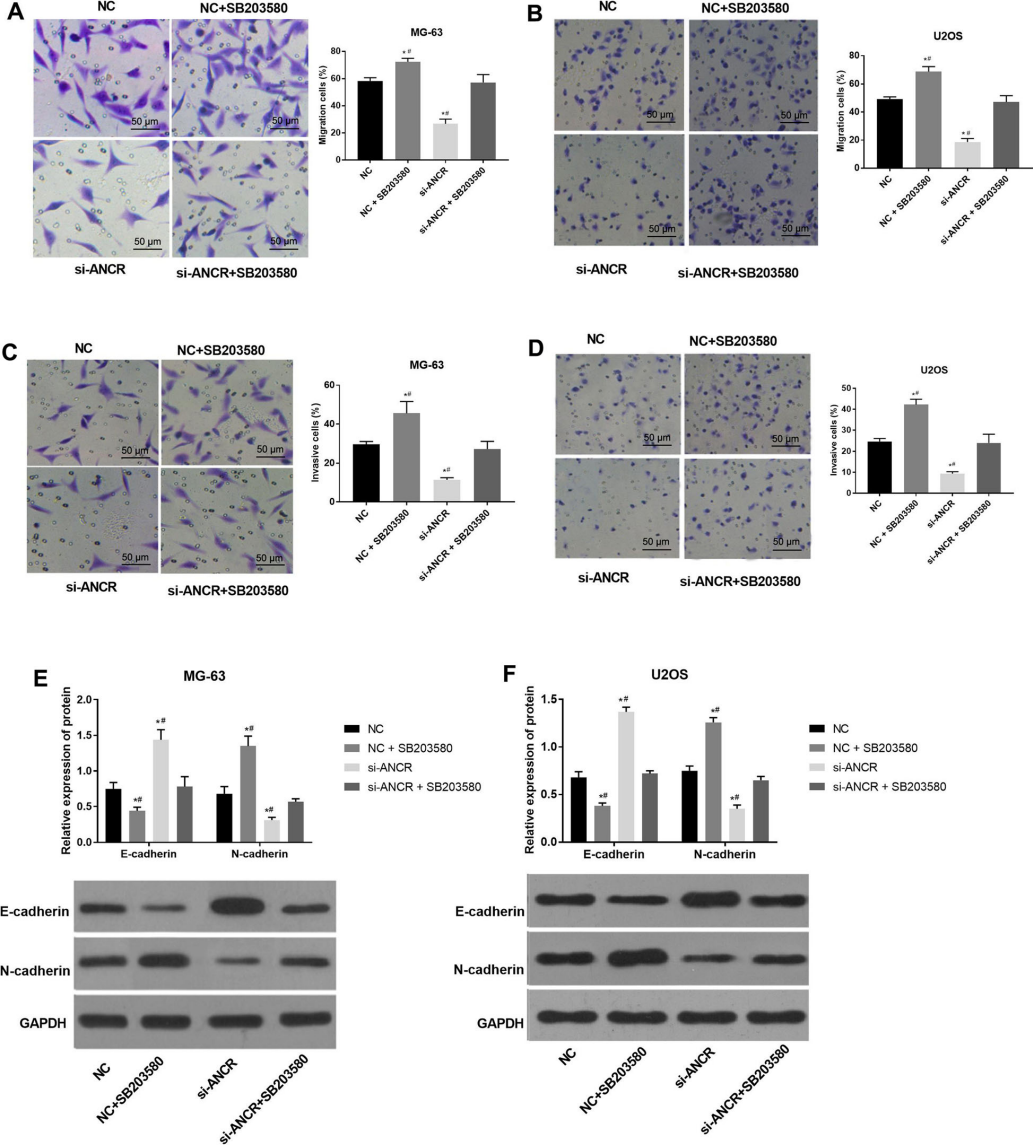
The migration and invasion of MG-63 and U2OS cells under the intervention of SB203580 (p38MAPK inhibitor) detected by Transwell assay and Western blot. **a** the percentage of migratory MG-63 cells (%) (*N* = 3); **b** the percentage of migratory U2OS cells (%) (*N* = 3); **c** the percentage of invasive MG-63 cells (%) (*N* = 3); **d** the percentage of invasive U2OS cells (%) (*N* = 3); **e** the expression of E-cadherin and N-cadherin in MG-63 cells at the protein level (*N* = 3); **f** the expression of E-cadherin and N-cadherin in U2OS cells at the protein level (*N* = 3). si-ANCR, cells transfected with siRNA-ANCR for 48 h; si-ANCR + SB203580, cells transfected with siRNA-ANCR and SB203580 (50 μmol/L) for 48 h; NC, cells transfected with siRNA negative control for 48 h; NC + SB203580, cells transfected with siRNA negative control and SB203580 (50 μmol/L) for 48 h. *, *P* < 0.05 vs. NC; #, *P* < 0.05 vs. si-ANCR + SB203580

In order to identify whether the effects of ANCR on cell migration and invasion are associated with MAPK signalling, SB203580 was used to inhibit the MAPK signalling pathway in MG-63 and U2OS cells. The Transwell assay showed that the percentage of cell migration was significantly increased upon treatment with SB203580 in both NC and si-ANCR groups (*P* < 0.05) (Fig. 5a and b). The percentage of invasive cells in NC and si-ANCR groups was also significantly increased upon treatment with SB203580 (*P* < 0.05) (Fig. 5c and d). The expression of migration and invasion-related proteins was further determined by western blot. SB203580 significantly down-regulated E-cadherin and up-regulated N-cadherin at the protein level (*P* < 0.05) (Fig. 5e and f). The above findings suggest that the inhibition of the p38MAPK signalling in OS cells can reduce si-ANCR-induced inhibition of cell migration and invasion.

## Discussion

OS is a bone sarcoma associated with a high level of genomic instability and high propensity for metastasis [24]. Many studies have shown that lncRNAs play a key regulatory role in cell proliferation, differentiation, senescence, and carcinogenesis [25–27]. Evidence has shown that cancer-associated lncRNAs exhibit carcinogenic or inhibitory effects on cancers, and that these lncRNAs may also be used as potential biomarkers or therapeutic targets [28, 29]. Therefore, we chose lncRNA-ANCR as the research focus of this study, and analysed the regulatory effect of ANCR in OS as well as the mechanism involving MAPK signalling pathway. Our results showed that ANCR silencing in OS cells could suppress cell proliferation, migration, and invasion via activating the p38MAPK signalling pathway.

In our research, ANCR expression was determined in OS cells and tissues, and a relatively high ANCR expression was observed. This finding is consistent with a previous study showing that ANCR expression is increased in OS tissues and cells [14]. Then, ANCR was down-regulated in OC cells through 48 h of transfection with si-ANCR and the cell biological processes were analysed. We found that ANCR silencing inhibited cell proliferation, migration and invasion, up-regulated E-cadherin, and down-regulated N-cadherin in MG-63 and U2OS cells. Although the prognosis of patients with OS improved significantly with chemotherapy, a proportion of those patients do not respond well to chemotherapeutic drugs. In addition, distant metastasis and local recurrence following surgical resection and chemotherapy also lead to poor prognosis [30]. The transmembrane glycoprotein E-cadherin plays an important role in homophilic cell interactions. Loss of E-cadherin function may promote tumour progression through inducing motile and invasive phenotypes [31, 32]. The adhesion protein N-cadherin is involved in the regulation of bone formation and embryonic development [33]. Previous research has proved that E-cadherin and N-cadherin are key regulatory molecules in cell adhesion, migration as well as tumour invasiveness [34]. In agreement with our study, Zhang et al. have proved that cell proliferation, migration, and invasion were suppressed by ANCR silencing in OS cells [14].

The MAPK family exerts a key regulatory role in the OS cell survival and angiogenesis [35]. Here, the effect of ANCR silencing on the biological function of OS cells was found to be associated with the activation of the p38MAPK signalling. The si-ANCR-induced anti-metastasis effect was eliminated by blocking p38MAPK. It was previously reported that the increase in p38MAPK levels inhibits the formation of metastatic tumours [36]. In addition, p38MAPK positively regulated the expression of E-cadherin, Snail, and Slug proteins [37, 38]. Another study suggested that the inhibition of the MAPK pathway can induce EZH2 expression in human breast cancer cells [39]. Furthermore, silencing of lncRNA-ANCR down-regulated EZH2 in OS at the mRNA level of [14]. Therefore, we speculate that ANCR silencing may influence the function of OS cells by activating the p38MAPK signalling pathway.

## Conclusions

As a conclusion, lncRNA-ANCR was up-regulated in OS. Silencing of lncRNA-ANCR suppressed cell proliferation, migration, and invasion through activation of the p38MAPK signalling pathway, which has an important clinical significance in the treatment of OS.

## Data Availability

All data generated or analyzed during this study are included in this published article [and its supplementary information files].

## Abbreviations

lncRNA: Long non-coding RNA
NC: Negative control
OS: Osteosarcoma

## References

[1] Tang J, Shen L, Yang Q, Zhang C (2014). Overexpression of metadherin mediates metastasis of osteosarcoma by regulating epithelial-mesenchymal transition. Cell Prolif.

[2] Hu J, Lv G, Zhou S, Zhou Y, Nie B, Duan H, Zhang Y, Yuan X (2015). The Downregulation of MiR-182 is associated with the growth and invasion of osteosarcoma cells through the regulation of TIAM1 expression. PLoS One.

[3] Yan K, Gao J, Yang T, Ma Q, Qiu X, Fan Q, Ma B (2012). MicroRNA-34a inhibits the proliferation and metastasis of osteosarcoma cells both in vitro and in vivo. PLoS One.

[4] Sampson VB, Yoo S, Kumar A, Vetter NS, Kolb EA (2015). MicroRNAs and potential targets in osteosarcoma: review. Front Pediatr.

[5] Isakoff MS, Bielack SS, Meltzer P, Gorlick R (2015). Osteosarcoma: current treatment and a collaborative pathway to success. J Clin Oncol.

[6] Ando K, Heymann MF, Stresing V, Mori K, Redini F, Heymann D (2013). Current therapeutic strategies and novel approaches in osteosarcoma. Cancers.

[7] Li HY, Zhang J, Sun LL, Li BH, Gao HL, Xie T, Zhang N, Ye ZM (2015). Celastrol induces apoptosis and autophagy via the ROS/JNK signaling pathway in human osteosarcoma cells: an in vitro and in vivo study. Cell Death Dis.

[8] Namlos HM, Meza-Zepeda LA, Baroy T, Ostensen IH, Kresse SH, Kuijjer ML, Serra M, Burger H, Cleton-Jansen AM, Myklebost O (2012). Modulation of the osteosarcoma expression phenotype by microRNAs. PLoS One.

[9] Jalali S, Bhartiya D, Lalwani MK, Sivasubbu S, Scaria V (2013). Systematic transcriptome wide analysis of lncRNA-miRNA interactions. PLoS One.

[10] Zhou M, Zhao H, Wang Z, Cheng L, Yang L, Shi H, Yang H, Sun J (2015). Identification and validation of potential prognostic lncRNA biomarkers for predicting survival in patients with multiple myeloma. J Exp Clin Cancer Res.

[11] Li Z, Hou P, Fan D, Dong M, Ma M, Li H, Yao R, Li Y, Wang G, Geng P (2017). The degradation of EZH2 mediated by lncRNA ANCR attenuated the invasion and metastasis of breast cancer. Cell Death Differ.

[12] Jia Q, Jiang W, Ni L (2015). Down-regulated non-coding RNA (lncRNA-ANCR) promotes osteogenic differentiation of periodontal ligament stem cells. Arch Oral Biol.

[13] Min L, Hong S, Duan H, Zhou Y, Zhang W, Luo Y, Shi R, Tu C (2016). Antidifferentiation noncoding RNA regulates the proliferation of osteosarcoma cells. Cancer Biother Radiopharm.

[14] Zhang F, Peng H (2017). LncRNA-ANCR regulates the cell growth of osteosarcoma by interacting with EZH2 and affecting the expression of p21 and p27. J Orthop Surg Res.

[15] Feng YL, Yin YX, Ding J, Yuan H, Yang L, Xu JJ, Hu LQ (2017). Alpha-1-antitrypsin suppresses oxidative stress in preeclampsia by inhibiting the p38MAPK signaling pathway: an in vivo and in vitro study. PLoS One.

[16] Fernandez F, Soon I, Li Z, Kuan TC, Min DH, Wong ES, Demidov ON, Paterson MC, Dawe G, Bulavin DV (2012). Wip1 phosphatase positively modulates dendritic spine morphology and memory processes through the p38MAPK signaling pathway. Cell Adhes Migr.

[17] Gaundar SS, Bendall LJ (2010). The potential and limitations of p38MAPK as a drug target for the treatment of hematological malignancies. Curr Drug Targets.

[18] Chen HJ, Lin CM, Lee CY, Shih NC, Peng SF, Tsuzuki M, Amagaya S, Huang WW, Yang JS (2013). Kaempferol suppresses cell metastasis via inhibition of the ERK-p38-JNK and AP-1 signaling pathways in U-2 OS human osteosarcoma cells. Oncol Rep.

[19] Cheng DD, Zhu B, Li SJ, Yuan T, Yang QC, Fan CY (2017). Down-regulation of RPS9 inhibits osteosarcoma cell growth through inactivation of MAPK signaling pathway. J Cancer.

[20] Zhang Y, Deng X, Lei T, Yu C, Wang Y, Zhao G, Luo X, Tang K, Quan Z, Jiang D (2017). Capsaicin inhibits proliferation and induces apoptosis in osteosarcoma cell lines via the mitogenâ ‘activated protein kinase pathway. Oncol Rep.

[21] Zhu J, Yu W, Liu B, Wang Y, Shao J, Wang J, Xia K, Liang C, Fang W, Zhou C (2017). Escin induces caspase-dependent apoptosis and autophagy through the ROS/p38 MAPK signalling pathway in human osteosarcoma cells in vitro and in vivo. Cell Death Dis.

[22] Wen L, Liang C, Chen E, Chen W, Liang F, Zhi X, Wei T, Xue F, Li G, Yang Q (2016). Regulation of multi-drug resistance in hepatocellular carcinoma cells is TRPC6/calcium dependent. Sci Rep.

[23] Li Z, Ning S, Zhang S (2014). Tumor PHD2 expression is correlated with clinical features and prognosis of patients with HCC receiving liver resection. Medicine.

[24] Moriarity BS, Otto GM, Rahrmann EP, Rathe SK, Wolf NK, Weg MT, Manlove LA, LaRue RS, Temiz NA, Molyneux SD (2015). A sleeping beauty forward genetic screen identifies new genes and pathways driving osteosarcoma development and metastasis. Nat Genet.

[25] Guttman M, Donaghey J, Carey BW, Garber M, Grenier JK, Munson G, Young G, Lucas AB, Ach R, Bruhn L (2011). lincRNAs act in the circuitry controlling pluripotency and differentiation. Nature.

[26] Du Z, Fei T, Verhaak RG, Su Z, Zhang Y, Brown M, Chen Y, Liu XS (2013). Integrative genomic analyses reveal clinically relevant long noncoding RNAs in human cancer. Nat Struct Mol Biol.

[27] Zhang Z, Zhu Z, Watabe K, Zhang X, Bai C, Xu M, Wu F, Mo YY (2013). Negative regulation of lncRNA GAS5 by miR-21. Cell Death Differ.

[28] Li Q, Su Z, Xu X, Liu G, Song X, Wang R, Sui X, Liu T, Chang X, Huang D (2012). AS1DHRS4, a head-to-head natural antisense transcript, silences the DHRS4 gene cluster in cis and trans. Proc Natl Acad Sci U S A.

[29] Gupta RA, Shah N, Wang KC, Kim J, Horlings HM, Wong DJ, Tsai MC, Hung T, Argani P, Rinn JL (2010). Long non-coding RNA HOTAIR reprograms chromatin state to promote cancer metastasis. Nature.

[30] Kobayashi E, Hornicek FJ, Duan Z (2012). MicroRNA involvement in osteosarcoma. Sarcoma.

[31] Canel M, Serrels A, Frame MC, Brunton VG (2013). E-cadherin-integrin crosstalk in cancer invasion and metastasis. J Cell Sci.

[32] Hu QP, Kuang JY, Yang QK, Bian XW, Yu SC (2016). Beyond a tumor suppressor: soluble E-cadherin promotes the progression of cancer. Int J Cancer.

[33] Xu L, Meng F, Ni M, Lee Y, Li G (2013). N-cadherin regulates osteogenesis and migration of bone marrow-derived mesenchymal stem cells. Mol Biol Rep.

[34] Hazan RB, Phillips GR, Qiao RF, Norton L, Aaronson SA (2000). Exogenous expression of N-cadherin in breast cancer cells induces cell migration, invasion, and metastasis. J Cell Biol.

[35] Zhang Y, Deng X, Lei T, Yu C, Wang Y, Zhao G, Luo X, Tang K, Quan Z, Jiang D (2017). Capsaicin inhibits proliferation and induces apoptosis in osteosarcoma cell lines via the mitogenactivated protein kinase pathway. Oncol Rep.

[36] del Barco BI, Nebreda AR (2012). Roles of p38 MAPKs in invasion and metastasis. Biochem Soc Trans.

[37] Thiery JP, Acloque H, Huang RY, Nieto MA (2009). Epithelial-mesenchymal transitions in development and disease. Cell.

[38] Kang HM, Park BS, Kang HK, Park HR, Yu SB, Kim IR (2018). Delphinidin induces apoptosis and inhibits epithelial-to-mesenchymal transition via the ERK/p38 MAPK-signaling pathway in human osteosarcoma cell lines. Environ Toxicol.

[39] Nishioka C, Ikezoe T, Yang J, Yokoyama A (2015). Tetraspanin family member, CD82, regulates expression of EZH2 via inactivation of p38 MAPK signaling in leukemia cells. PLoS One.

